# Sympathetic vasomotor outflow and blood pressure increase during exercise with expiratory resistance

**DOI:** 10.14814/phy2.12421

**Published:** 2015-05-27

**Authors:** Keisho Katayama, Yuka Itoh, Mitsuru Saito, Teruhiko Koike, Koji Ishida

**Affiliations:** 1 Research Center of Health, Physical Fitness and Sports, Nagoya UniversityNagoya, Japan; 2 Graduate School of Medicine, Nagoya UniversityNagoya, Japan; 3 Faculty of Psychological and Physical Science, Aichigakuin UniversityNisshin, Japan

**Keywords:** Dynamic leg exercise, metaboreflex, respiratory muscle, sympathetic outflow

## Abstract

The purpose of the present study was to elucidate the effect of increasing expiratory muscle work on sympathetic vasoconstrictor outflow and arterial blood pressure (BP) during dynamic exercise. We hypothesized that expiratory muscle fatigue would elicit increases in sympathetic vasomotor outflow and BP during submaximal exercise. The subjects performed four submaximal exercise tests; two were maximal expiratory pressure (PE_max_) tests and two were muscle sympathetic nerve activity (MSNA) tests. In each test, the subjects performed two 10-min exercises at 40% peak oxygen uptake using a cycle ergometer in a semirecumbent position [spontaneous breathing for 5 min and voluntary hyperpnoea with and without expiratory resistive breathing for 5 min (breathing frequency: 60 breaths/min, inspiratory and expiratory times were set at 0.5 sec)]. PE_max_ was estimated before and immediately after exercises. MSNA was recorded via microneurography of the right median nerve at the elbow. PE_max_ decreased following exercise with expiratory resistive breathing, while no change was found without resistance. A progressive increase in MSNA burst frequency (BF) appeared during exercise with expiratory resistance (MSNA BF, without resistance: +22 ± 5%, with resistance: +44 ± 8%, *P* < 0.05), accompanied by an augmentation of BP (mean BP, without resistance: +5 ± 2%, with resistance: +29 ± 5%, *P* < 0.05). These results suggest that an enhancement of expiratory muscle activity leads to increases in sympathetic vasomotor outflow and BP during dynamic leg exercise.

## Introduction

High-intensity whole body exercise induces inspiratory muscle (diaphragm) fatigue (Johnson et al. 1993; Romer and Polkey 2008). In healthy humans, this exercise-induced inspiratory muscle fatigue does not limit the hyperventilatory response to exercise. However, it has been thought that the fatiguing inspiratory muscle affects cardiovascular regulation and blood flow distribution during exercise (Harms et al. 1998; Dempsey et al. 2006, 2008). Voluntary breathing against inspiratory resistance at rest and during exercise augments an increase in muscle sympathetic nerve activity (MSNA), that is, sympathetic vasomotor outflow, with corresponding increase in arterial blood pressure (BP) (St Croix et al. 2000; Sheel et al. 2001; Katayama et al. 2012, 2013). This sympathoexcitation occurs through an inspiratory muscle fatigue-induced metaboreflex (Hill 2000).

Similar to the inspiratory muscle, expiratory muscle fatigue occurs following exercise (Loke et al. 1982; Fuller et al. 1996; Taylor et al. 2006). Fatiguing expiratory muscles could also affect cardiovascular regulation. Indeed, high-intensity contractions of expiratory muscles in the resting state cause increases in MSNA and BP (Derchak et al. 2002). Recently, Athanasopoulos et al. (2010) revealed an increase in blood flow in the expiratory muscle and a concomitant decrease in quadriceps muscle blood flow during leg exercise with expiratory muscle loading. Clinically, respiratory muscle fatigue could play an important role in cardiovascular regulation during exercise, because inspiratory and expiratory respiratory muscle works are enhanced during exercise in patients with chronic obstructive pulmonary disease (COPD) and chronic heart failure (CHF) (Sullivan et al. 1988; Musch 1993; Amann et al. 2010). Significant expiratory (abdominal) muscle fatigue occurs following exercise in patients with COPD (Hopkinson et al. 2010), although interindividual variability exists (Bachasson et al. 2013). Verges et al. (2007) reported that expiratory muscle fatigue impairs exercise performance as reported for inspiratory muscle fatigue. From these studies, it is assumed that an increase in expiratory muscle work activates metaboreflex resulting in increased sympathetic vasomotor outflow and thereby compromising blood flow and oxygen delivery during exercise. However, to the best of our knowledge, direct recording of sympathetic vasomotor outflow and cardiovascular variables during dynamic exercise with expiratory resistance has not been performed.

The purpose of the present study was to elucidate the effect of increasing expiratory muscle work on sympathetic vasoconstrictor outflow and BP during dynamic exercise. We recorded MSNA and cardiovascular variables during a leg-cycling exercise with expiratory resistance. We hypothesized that expiratory muscle fatigue would elicit increases in sympathetic vasomotor outflow and BP during submaximal exercise.

## Methods

### Subjects

Nine healthy males participated in the study; eight of these completed the study [means ± SE: age = 23 ± 1 year, height = 172 ± 2 cm, body mass = 68 ± 3 kg, forced vital capacity = 4.5 ± 0.1 L, forced expiratory volume in 1s = 4.0 ± 0.1 L, 89 ± 1%, maximal inspiratory pressure (PI_max_) = −133 ± 6 cmH_2_O]. All were nonsmokers with no history of respiratory or cardiovascular disease. Subjects were informed about the experimental procedures and potential risks involved, and written consent was obtained. This study was approved by the human research committee of the Research Center of Health, Physical Fitness and Sports, Nagoya University.

## Experimental procedure

At the preliminary visit, the subjects were instructed on how to laterally extend both arms and how to hold their arms during leg-cycle exercise using an electromechanically braked ergometer in a semirecumbent position (Aerobike 75XL, Combi, Tokyo, Japan). Subjects reported to the laboratory on at least four occasions at the same time of day, and each visit was separated by 1 week.

On day 1, the subjects performed an incremental exercise test using the ergometer (maximal exercise test) to determine peak oxygen uptake (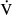
O_2peak_). The exercise test began at an initial power output of 90 W, and the workload was increased by 15 W every minute until exhaustion. The pedaling rate was maintained at 60 rpm with the aid of a metronome. Minute expired ventilation (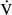
E), oxygen uptake (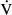
O_2_), heart rate (HR), and arterial oxygen saturation (SpO_2_) were recorded during the test and were averaged every 30s afterward. The highest 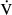
O_2_ value obtained was used as 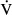
O_2peak_. Then, workload at 40% 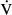
O_2peak_ was calculated for the submaximal exercise test.

On day 2, subjects were again instructed on how to hold their right arm during submaximal exercise. Additionally, the subjects practiced controlling their breaths during exercise with or without the expiratory resistance by means of oscilloscope. The subjects also practiced measuring maximal expiratory pressure (PE_max_) before and immediately after exercise.

**Figure 1:**
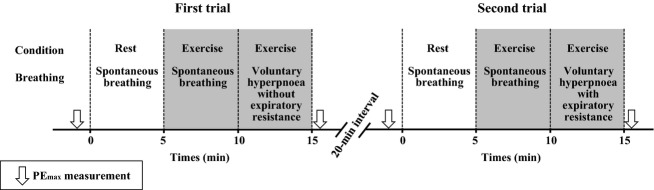
Time course of the experiment (PE_max_ test). MSNA test was done as the same of PE_max_ test, except PE_max_ measurement.

On day 3, the subjects carried out two submaximal exercise tests with PE_max_ measurement (PE_max_ test), as shown in Fig.1. The PE_max_ tests were performed to assess expiratory muscle fatigue using expiratory resistance during exercise. The subjects rested for 30 min, and a PE_max_ measurement was taken before exercise. The subjects then rested for 5 min (rest-spontaneous breathing). Respiratory and cardiovascular variables were measured throughout the experiment. Submaximal exercise was then carried out for 10 min, and exercise intensity was set at 40% 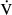
O_2peak_. The pedaling rate was kept at 60 rpm with the aid of a metronome. The subjects breathed spontaneously over the first 5 min of exercise (exercise-spontaneous breathing). During the next 5 min, the subjects were asked to control their breath with or without expiratory resistance (exercise-voluntary hyperpnoea): breathing frequency (fb) was maintained at 60 breaths/min and the inspiratory and expiratory time of one breath cycle was set at 0.5 s via auditory feedback from the metronome. Tidal volume (VT) was regulated at twice the resting VT via visual feedback from an oscilloscope marked with target VT levels (Sheel et al. 2001; Katayama et al. 2012, 2013). End-tidal partial pressure of CO_2_ (PETCO_2_) was maintained within ±3 mmHg of the level during exercise-spontaneous breathing by adding CO_2_ to the inspired air. The subjects were asked to report their rate of perceived exertion (RPE) (Borg 1982) for dyspnea during the last minute of each 5-min exercise, that is, the exercise-spontaneous breathing and the exercise-voluntary hyperpnoea. Immediately after exercise, PE_max_ measurement was performed. The procedure was repeated twice; that is, the exercise-voluntary hyperpnoea with or without expiratory resistance, with a 20-min interval between trials. In a preliminary study, we confirmed that the reduced PE_max_ and the enhanced MSNA after exercise with expiratory resistance did not return to pre-exercise levels within 20 min. Therefore, the exercise-voluntary hyperpnoea without expiratory resistance was performed first (First trial), and the exercise-voluntary hyperpnoea with expiratory resistance was done second (Second trial) (Fig.1). The resistance device was connected to the expiratory side via a tube. Expiratory resistance during exercise was set at 20% PE_max_, because we confirmed that several subjects could not keep target VT levels for 5 min when expiratory resistance was set at 30% PE_max_.

On day 4, the subjects performed the two submaximal exercise tests with MSNA measurement (MSNA test) to elucidate the influence of expiratory muscle fatigue on MSNA during exercise. The procedures and measurements of respiratory and cardiovascular parameters were identical to those of the PE_max_ test (Fig.1).

Initially, a total of nine subjects entered the study. MSNA recordings were completed for six of these, while the remaining three failed to record MSNA during exercise because of displacement of the electrodes from the muscle sympathetic nerve or bursts as a result of movement of the arm or body. In these three subjects, the MSNA test was repeated after a break of at least 1 month, at that point, MSNA recordings were completed in two of the three subjects. Consequently, eight subjects from whom we obtained nerve recordings were used in the analysis.

### Expiratory muscle strength

PE_max_, as an indicator of expiratory muscle strength, was measured using a handheld mouth pressure meter (AAM377; Minato Ikagaku, Osaka, Japan) connected to a computerized spirometry system (AS-507; Minato Ikagaku). All measurements were taken from total lung capacity, the subjects brought their hand to their cheeks and pressed forcefully (Suzuki et al. 1991; Fuller et al. 1996). For each measurement, five trials were completed, and the highest of three measurements with less than 5% variability was averaged and used as PE_max_ (Suzuki et al. 1991). If three measurements with less than 5% were not obtained within five trials, the procedure was repeated.

### Respiratory variables

Subjects breathed through a mouthpiece with their nose occluded. The mouthpiece was attached to a hot wire flowmeter (RF-H; Minato Ikagaku), which was connected to a device equipped with a one-way low resistance valve. The dead space in this ventilatory system was ~130 mL. The flow signal from the flowmeter was connected to an oscilloscope, which indicated the target VT as a horizontal line for visual feedback. This system was similar to that in our previous studies (Katayama et al. 2012, 2013). Sample gas was drawn through a sampling tube connected to the mouthpiece in order to measure end-tidal O_2_ fraction (FETO_2_) and end-tidal CO_2_ fraction (FETCO_2_) by means of a gas analyzer (MG-360; Minato Ikagaku). 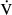
E, VT, fb, and 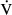
O_2_ were determined using an online system with a mixing chamber, as in our previous studies (Katayama et al. 2011, 2012, 2013). Expired gas volume was measured by a Fleisch pneumotachometer (PN-230; Arco Systems, Chiba, Japan), which was connected to the expiratory side of the valve via tube. Sample gas was drawn through a sampling tube inserted into the pneumotachometer to measure expired gas fractions. The expired gas fractions were analyzed using a mass spectrometer (ARCO-1000; Arco Systems) that was calibrated and confirmed before each test. Breath-by-breath data were analyzed continuously using customized computer software (PC-9821Ra40; NEC, Tokyo, Japan). SpO_2_ was measured using a finger pulse oximeter (Biox 3740; Ohmeda, Madison, WI). The signals from the flowmeter, gas analyzer, and pulse oximeter were sampled at a frequency of 200 Hz through an analog-to-digital converter (CSI-320416; Interface, Hiroshima, Japan) and were stored in a computer (CF-F8; Panasonic, Osaka, Japan). The signals were analyzed afterward using our own computer software.

### Cardiovascular variables

An electrocardiogram (ECG) was measured using a three-lead electrocardiograph (AB-621; Nihon Koden, Tokyo, Japan), and HR was calculated from each R-R interval obtained from the ECG. Beat-to-beat arterial BP was acquired using finger photoplethysmography from the middle finger of the left hand (Finometer, Finapres Medical Systems BV, Amsterdam, The Netherlands). ECG and BP signals were sampled and analyzed using a method similar to that for respiratory variables. Arterial systolic and diastolic BP (SBP and DBP) were determined from the BP wave form signal, and mean arterial BP (MBP) was calculated using the following equation: MBP = (SBP-DBP) / 3+ DBP.

### MSNA

Multiunit muscle sympathetic nerve discharges were recorded using the microneurographic technique. A recording system similar to that in our previous studies (Katayama et al. 2011, 2012, 2013, 2014) was utilized. A tungsten microelectrode with shaft diameter of 0.1 mm (impedance 1–5 MΩ) was inserted manually by an experimenter into the right median nerve at the cubital fossa (Saito et al. 1993; Katayama et al. 2011, 2012, 2013, 2014). The right arm was fixed using equipment to prevent arm movement artifacts during the leg cycling exercise. The electrode was adjusted until MSNA was recorded after insertion. Identification of MSNA was based on the following criteria: spontaneous burst discharge synchronized with the heartbeat and enhanced by Valsalva maneuver or breath holding, but showing no change in response to sensory stimuli, such as a loud noise or cutaneous touch (Delius et al. 1972; Vallbo et al. 1979; Fagius and Wallin 1980). Furthermore, we asked the subjects to hold their breath to identify MSNA at the middle phase during the exercise-spontaneous breathing and after exercise (at least 5 sec). The neurogram was fed to a differential amplifier and amplified 100,000 times through a band-pass filter (700–2000 Hz). The neurogram was full-wave rectified and integrated by a capacitance-integrated circuit with a time constant of 0.1 sec. The mean voltage neurogram was continuously digitized through an analog-to-digital converter with a sampling frequency of 200 Hz for storage on a computer in a manner similar to that for used respiratory and cardiovascular variables. MSNA bursts were identified from the mean voltage neurogram using a customized computer program-assisted inspection (Katayama et al. 2011, 2012, 2013, 2014), which accounted for the latency from the ECG-R wave to the sympathetic burst (Fagius and Wallin 1980). MSNA was quantified as burst frequency (BF, bursts/min) and burst incidence (BI, bursts/100HR) (Katayama et al. 2011, 2012, 2013, 2014). Since electromyographic efferent and afferent nerve activities altered the baseline of the integrated neurogram during dynamic leg cycling in most recordings (Saito et al. 1993; Katayama et al. 2011, 2012, 2013, 2014), we could not calculate MSNA burst amplitude and total activity.

### Statistical analysis

Values are expressed as means ± SE. The respiratory and cardiovascular variables, and MSNA BF values were averaged every 1 min throughout the experiment. The assumption of normal distribution for all data was verified using a Kolmogorov-Smirnov test. Changes in parameters during the experiment in each trial were analyzed using a Dunnett's test, that is, vs. at 5 min at the rest-spontaneous breathing or vs. at 5 min during the exercise-spontaneous breathing. Comparisons of parameters between the nonresistance and resistance trials were performed using paired *t*-test (parametric test) when the distribution was regular. When the distribution was not regular, a Wilcoxon test (nonparametric test) was used. The StatView (5.0; SAS Institute, Tokyo, Japan) and the SPSS (11.5; SPSS, Tokyo, Japan) statistical packages were used for the analyses. A *P* < 0.05 was considered to indicate significance.

## Results

### Maximal exercise test

Cardiorespiratory variables and workload at exhaustion during maximal exercise test are as follows: 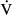
O_2_ = 3.1 ± 0.1 L/min, 46 ± 2 mL/kg/min, 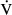
E = 122 ± 8 L/min, HR = 185 ± 1 beats/min, SpO_2_ = 96 ± 4%, and workload = 255 ± 4W.

### Submaximal exercise test

#### Baseline descriptive data

No significant differences were found in any of the respiratory and cardiovascular variables throughout the experiment between the PE_max_ and MSNA tests. Therefore, we report only the data during the MSNA test. No differences in these variables were found at the rest-spontaneous breathing between the first and second trials. Workload during submaximal exercise was 75 ± 5W.

#### Expiratory muscle strength

In the first trial, PE_max_ was unchanged after exercise (182 ± 12 to 183 ± 13 cmH_2_O). On the contrary, a significant reduction of PE_max_ appeared in the second trial (188 ± 13 to 158 ± 12 cmH_2_O, *P* < 0.05).

#### RPE

RPE dyspnea scores increased from the exercise-spontaneous breathing to the exercise-voluntary hyperpnoea in the first (10.5 ± 0.3 to 11.8 ± 0.3, *P* < 0.05) and second (10.5 ± 0.3 to 14.0 ± 0.5, *P* < 0.05) trials. RPE dyspnea in the exercise-voluntary hyperpnoea with expiratory resistance was higher (*P* < 0.05) than the hyperpnoea without resistance.

#### Respiratory variables

**Figure 2:**
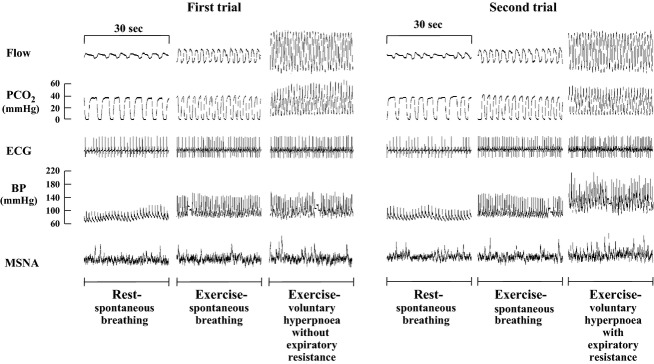
Representative records of flow, PCO_2_, ECG, BP, and MSNA in the first and second trials.

Representative flow and partial pressure of CO_2_ (PCO_2_) during the first and second trials are shown in Fig.2, and mean values of respiratory parameters were shown in Table1. 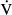
E, VT, and fb and PETCO_2_ increased (*P* < 0.05) during the exercise-spontaneous breathing, and 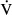
E, and fb increased (*P* < 0.05) further during the exercise-voluntary hyperpnoea. A small but significant increase in SpO_2_ appeared during the exercise-voluntary hyperpnoea. There were no significant differences in all respiratory parameters between the first and second trials throughout the experiment.

**Table 1:** Respiratory variables in the MSNA test.

	Trials	Rest-spontaneous breathing	Exercise- spontaneous breathing	Exercise- voluntary hyperpnoea	
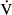 E (L/min)	First	10.2 ± 0.4	27.8 ± 1.1^1^	Without resistance	72.1 ± 2.3^1^^,^^3^
	Second	11.8 ± 0.8	29.3 ± 1.5^2^	With resistance	70.4 ± 3.1^2^^,^^4^
VT (L)	First	0.61 ± 0.03	1.23 ± 0.09^1^	Without resistance	1.23 ± 0.04^1^
	Second	0.64 ± 0.02	1.32 ± 0.13^2^	With resistance	1.19 ± 0.06^2^
fb (breaths/min)	First	13.6 ± 1.4	24.1 ± 1.5^1^	Without resistance	59.6 ± 0.2^1^^,^^3^
	Second	14.4 ± 1.4	24.3 ± 2.4^2^	With resistance	59.8 ± 0.1^2^^,^^4^
PETO_2_ (mmHg)	First	105.2 ± 1.6	106.7 ± 1.7	Without resistance	134.0 ± 1.4^1^^,^^3^
	Second	108.0 ± 1.1	107.3 ± 2.0	With resistance	134.9 ± 1.3^2^^,^^4^
PETCO_2_ (mmHg)	First	44.4 ± 0.7	45.5 ± 0.6^1^	Without resistance	45.5 ± 0.6^1^
	Second	44.0 ± 0.8	45.8 ± 0.7^2^	With resistance	45.1 ± 0.7^2^
SpO_2_ (%)	First	97.3 ± 0.3	97.3 ± 0.3	Without resistance	98.7 ± 0.2^1^^,^^3^
	Second	97.7 ± 0.2	97.5 ± 0.2	With resistance	99.0 ± 0.3^2^^,^^4^

Values are mean ± SE. 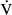
E, expired minute ventilation; VT, tidal volume; fb, breathing frequency; PETO_2_, end-tidal partial pressure of O_2_; PETCO_2_, end-tidal partial pressure of CO_2_; SpO_2_, arterial oxygen saturation.

1 Significant from Rest-spontaneous breathing in the first trial, *P* < 0.05.

2 Significant from Rest-spontaneous breathing in the second trial, *P* < 0.05.

3 Significant from Exercise-spontaneous breathing in the first trial, *P* < 0.05.

4 Significant from Exercise-spontaneous breathing in the second trial, *P* < 0.05.

#### Cardiovascular variables

**Figure 3:**
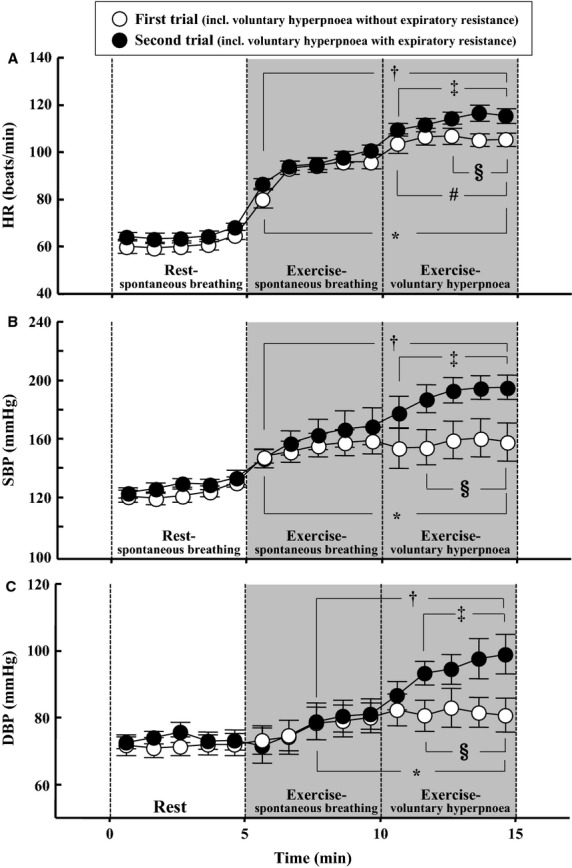
Changes in HR (A), SBP (B), and DBP (C) during the experiment. *P < 0.05 vs. at 5 min during rest-spontaneous breathing in the first trial. ^†^*P* < 0.05 vs. at 5 min during rest-spontaneous breathing in the second trial. ^#^*P* < 0.05 vs. at 5 min during exercise-spontaneous breathing in the first trial. ^‡^at 5 min during exercise-spontaneous breathing in the second trial. ^§^*P* < 0.05 between the first and second trials.

Typical recordings of ECG and BP are indicated in Fig.2. Mean values of HR, SBP, and DBP are shown in Fig.3. These variables increased (*P* < 0.05) during the exercise-spontaneous breathing in the first and second trials. Significant increases in HR, SBP, and DBP occurred during the exercise-voluntary hyperpnoea with expiratory resistance. During the exercise-voluntary hyperpnoea without expiratory resistance, a small but significant increase in HR occurred during, whereas SBP and DBP were unchanged. HR, SBP, and DBP during the latter part of the exercise-voluntary hyperpnoea with expiratory resistance were higher (*P* < 0.05) than those in the hyperpnoea without expiratory resistance.

#### MSNA

**Figure 4:**
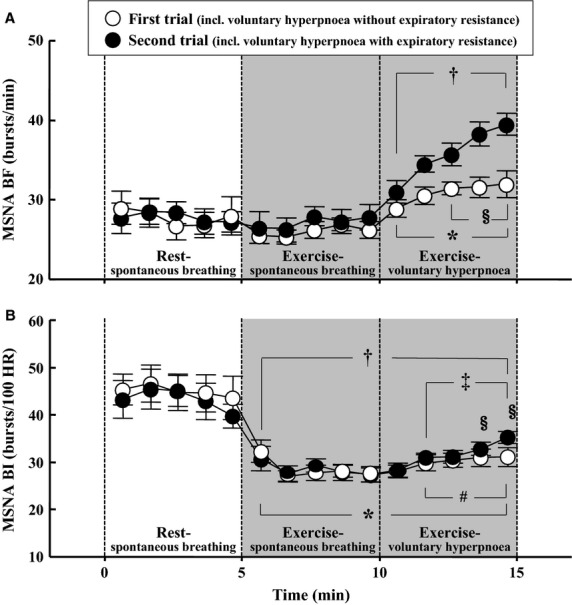
Changes in MSNA BF (A) and MSNA BI (B) during the experiment. *P < 0.05 vs. at 5 min during rest-spontaneous breathing in the first trial. ^†^*P* < 0.05 vs. at 5 min during rest-spontaneous breathing in the second trial. ^#^*P* < 0.05 vs. at 5 min during exercise-spontaneous breathing in the first trial. ^‡^at 5 min during exercise-spontaneous breathing in the second trial. ^§^*P* < 0.05 between the first and second trials.

Representative MSNA recordings are indicated in Fig.2, and mean MSNA BF and BI values are shown in Fig.4. MSNA BF did not change during the exercise-spontaneous breathing in both trials. A progressive increase in MSNA BF appeared during the exercise-voluntary hyperpnoea with expiratory resistance. MSNA BF exhibited a small but significant increase by voluntary hyperpnoea without expiratory resistance. The extent of MSNA BF during the latter part of the exercise-voluntary hyperpnoea with expiratory resistance was larger (*P* < 0.05) than the hyperpnoea without resistance. MSNA BI decreased significantly during the exercise-spontaneous breathing. During the exercise-voluntary hyperpnoea, MSNA BI increased in both with and without expiratory resistance, but the magnitude of an increase in MSNA BI was higher in the exercise-voluntary hyperpnoea with resistance.

## Discussion

The primary finding of the present study was that an increase in MSNA BF appeared during a leg-cycling exercise with expiratory resistive breathing, accompanied by an augmentation of BP. This finding supports our hypothesis that the expiratory muscle fatigue may lead to increase in sympathetic vasomotor outflow and BP during submaximal exercise. The results from this study should provide information regarding the significance of expiratory muscle activity on circulatory regulation during dynamic exercise.

### Effect of expiratory resistance on expiratory muscle strength

To estimate the effect of expiratory resistive breathing on expiratory muscle fatigue, we measured PE_max_, as an index of expiratory muscle strength, before and after submaximal exercise. Consequently, PE_max_ decreased significantly following the exercise-voluntary hyperpnoea with expiratory resistance, indicating that expiratory muscle fatigue had occurred. This result is in agreement with the data of previous studies, which demonstrated reductions in PE_max_ after resistive breathing (Suzuki et al. 1991; Orozco-Levi et al. 2001; Derchak et al. 2002). A reduction in the gastric pressure using magnetic nerve stimulation (Taylor et al. 2006) and large declines of electromyogram of the abdominal muscle during maximal expiratory efforts (Fuller et al. 1996) after high-intensity exercise have been reported. Abdominal muscles have a relatively high percentage of fast-twitch fibers (Johnson et al. 1973), and the oxidative capacity in abdominal muscles is lower than that in the diaphragm (Uribe et al. 1992). In addition, there is evidence that muscle endurance is lower in expiratory muscles compared with in inspiratory muscles (Gandevia et al. 1983). In our previous study (Katayama et al. 2012), inspiratory resistance during exercise was set at 29–40% PI_max_ under similar VT and fb, and the all subjects completed the inspiratory resistive breathing for 5 min. In contrast to this, in our preliminary study several subjects could not keep target VT levels for 5 min during exercise with expiratory resistive breathing at 30% PE_max_. From these observations, it is assumed that the expiratory muscle is more fatigable than the inspiratory muscle.

### Effect of expiratory resistance on MSNA and BP response during dynamic exercise

The exercise pressor reflex, the afferent arm of which is composed of type III and IV muscle afferents, plays a role in the cardiovascular adjustments (Adreani et al. 1997). The diaphragm has an abundance of type IV afferent fiber, and activity in the fiber increases during fatiguing diaphragm contractions (Hill 2000). Although there is no available data concerning innervation of the abdominal muscles of expiration by type III and IV afferents, many such fibers identify in the internal intercostal nerve that subserves the rib-cage expiratory muscles (Duron 1981). The ubiquity of these fibers in other skeletal muscle and the diaphragm suggests that type III and IV afferents are present in expiratory muscles (Derchak et al. 2002). At resting, it was revealed that high-intensity contractions of expiratory muscles cause an increase in MSNA (Derchak et al. 2002). In the present study, expiratory resistive breathing during leg cycling did cause a progressive increase in MSNA BF with an enhancement of BP. Collectively, it is plausible that a metaboreflex arising in the expiratory muscles is an important determinant of sympathetic vasomotor outflow and BP during submaximal exercise.

Other possible mechanisms affecting MSNA during exercise with expiratory resistive breathing should be considered. First, it is necessary to consider the increased central respiratory motor output (central command). The increased RPE dyspnea score indicates a higher central respiratory motor output during the exercise-voluntary hyperpnoea with expiratory resistance, and this may be a cause of the observed sympathoexcitation. Derchak et al. (2002) reported that an enhanced central respiratory motor output produced an increased HR but not MSNA and BP. HR is more vulnerable to central command influences (Victor et al. 1989). Similarly, St Croix et al. (2000) reported that an increase in central respiratory output led immediate an sustained increases in HR without significant effect on sympathetic outflow. However, in their studies (St Croix et al. 2000; Derchak et al. 2002), exceptional subjects demonstrated increases in MSNA during nonfatiguing, resistive breathing. Additionally, in the present study, a small but significant increase in MSNA BF was noted during the exercise-voluntary hyperpnoea without expiratory resistance (Fig.4). From these results, the effect of augmented central expiratory effort may involve the reasons for an increase in MSNA. Second, it has been demonstrated that heavy expiratory resistance causes not only PE_max_ reduction but also a decrease in PI_max_ as an index of inspiratory muscle fatigue (Suzuki et al. 1991; Haverkamp et al. 2001). However, expiratory resistance for short duration does not induce diaphragm fatigue (Derchak et al. 2002). Accordingly, it is conceivable that the inspiratory muscle fatigue did not appear and thereby would have only minor effects on MSNA via inspiratory muscle metaboreflex in the present study. Third, stimulation of mechanoreceptors in abdominal viscera may induce an increase in vagal afferent nerve traffic and BP, suggesting increased sympathetic vasomotor outflow (Gieroba et al. 1995). This is unlikely because the receptors respond mainly to distension, and the abdominal viscera were compressed, not distended, during expiratory efforts (Derchak et al. 2002). Fourth, cardiopulmonary baroreceptor reflex may affect the change in MSNA during exercise. During mild dynamic leg exercise, cardiopulmonary baroreflex could play an important role for sympathetic vasomotor outflow (Ray et al. 1993; Fadel and Raven 2012; Katayama et al. 2014). Enhanced intrathoracic pressure during expiratory resistance induces a large decrease in central blood volume, which may induce unloading cardiopulmonary receptors (Ray et al. 1993; Miller et al. 2007). Accordingly, cardiopulmonary baroreflex may be included as one of the reasons for the increased MSNA during exercise with expiratory resistance. Fifth, sympathetic vasomotor outflow is influence by mental stress (Callister et al. 1992). We asked the subjects to control their breath during exercise-voluntary hyperpnoea. This may affect MSNA response, especially with expiratory resistance, although we do not have enough data for how much stress the subjects had during the task. Finally, it is possible that pain may be associated with the increased MSNA during resistive breathing (St Croix et al. 2000; Sheel et al. 2001; Derchak et al. 2002). However, it was reported that exercise-induced increased MSNA disappeared after exercise while persistent muscle pain (Vissing et al. 1998). None of the subjects complained of pain following exercise with resistive breathing. Therefore, it is unlikely that such distress is related to the increased MSNA during the exercise-voluntary hyperpnoea with expiratory resistance.

### Technical considerations and limitations

Several technical considerations and limitations should be noted. One limitation was the use of PE_max_ as an index of expiratory muscle strength; we could not utilize a more definitive method to assess expiratory muscle function; that is., using gastric pressure response to magnetic stimulation (Kyroussis et al. 1996; Taylor et al. 2006). In the present study, five measurements were performed, and the highest of three measurements with less than 5% variability was averaged and used as PE_max_ (Suzuki et al. 1991). The maneuvers were repeated if three measurements with less than 5% were not obtained. Consequently, within-day coefficient of variation for PE_max_, which was calculated from the data before each trial, was 4.3%. Thus, PE_max_ data presented in the present study are valid and the PE_max_ values were comparable before and after exercise with or without expiratory resistance.

It is necessary to consider exercise intensity. As in our previous studies (Katayama et al. 2012, 2013, 2014), we utilized mild exercise intensity (40% 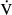
O_2peak_), because the percentage of successful MSNA recordings is high when the exercise intensity is mild. Another reason is that MSNA BF is not altered during leg cycling at 40% 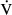
O_2peak_ exercise compared with when at rest (Katayama et al. 2012, 2013). In the present study, we confirmed this as shown in Fig.4. Therefore, we assumed that an increase in MSNA BF with expiratory resistive breathing would become apparent under these conditions.

MSNA was assessed as BF and BI in the present study. We could not estimate burst amplitude because electromyographic and efferent and afferent nerve activities altered the baseline of the integrated neurogram during dynamic leg cycling (Saito et al. 1993; Katayama et al. 2012, 2013). However, there is a positive correlation between BF and burst amplitudes (Mark et al. 1985) and parallel increases in BF and burst amplitude during exercise (Seals et al. 1991). Therefore, it seems reasonable to suppose that our MSNA BF values are valid and MSNA levels were comparable during leg cycling with or without resistive breathing.

### Perspective and significance

In this study, a progressive increase in MSNA BF appeared during exercise with expiratory resistance accompanied by an augmentation of BP. These results suggest that an enhancement of expiratory muscle activity leads to increases in sympathetic vasomotor outflow and BP during dynamic leg exercise. The results in this study support the idea that fatiguing respiratory muscle affects cardiovascular regulation during exercise (Harms et al. 1998; Dempsey et al. 2006, 2008). Fatigue of the expiratory muscle causes a sympathetically mediated vasoconstriction, similar to that described previously for fatiguing inspiratory muscle work (St Croix et al. 2000; Sheel et al. 2001; Katayama et al. 2012, 2013). In animal study, infusion of lactic acid into the deep circumflex iliac artery resulted in a reduction in hind limb blood flow (Rodman et al. 2003). Recently, it was revealed that blood flow in the expiratory muscle increases, quadriceps muscle blood flow decrease, during leg exercise with expiratory muscle loading (Athanasopoulos et al. 2010). A reduction in blood flow (oxygen transport) to working limb muscles would be expected to increase locomotor muscle fatigue (Taylor and Romer 2008). Indeed, it is confirmed that expiratory muscle fatigue impairs exercise performance (Verges et al. 2007). Clinically, respiratory muscle activity cold play an important role in limiting oxygen delivery during exercise, since it has been reported that patients with COPD and CHF have a large amount of respiratory muscle work (Sullivan et al. 1988; Musch 1993; Amann et al. 2010). Several studies demonstrated expiratory (abdominal) muscle fatigue following exercise in COPD patients (Hopkinson et al. 2010), although wide interindividual variability exists (Bachasson et al. 2013). Therefore, it is assumed that vasoconstriction, which is induced by sympathoexcitation via expiratory fatigue-induced metaboreflex during exercise, affects locomotor oxygen transport and consequent locomotor muscle fatigue and exercise tolerance in patients.
